# Outbreak of Coronavirus Disease 2019 (COVID‑19) in Pakistan: Psychological impact and coping strategies of Health Care Professionals

**DOI:** 10.12669/pjms.36.7.2988

**Published:** 2020

**Authors:** Khola Noreen, Muhammad Umar, Syed Arshad Sabir, Rehana Rehman

**Affiliations:** 1 Dr. Khola Noreen Assistant Professor Community Medicine Rawalpindi Medical University, Rawalpindi, Pakistan; 2 Prof Dr. Muhammad Umar Vice Chancellor Rawalpindi Medical University, Rawalpindi, Pakistan; 3 Prof. Dr. Syed Arshad Sabir Dean Public Health & Community Medicine Rawalpindi Medical University, Rawalpindi, Pakistan; 4 Dr. Rehana Rehman Department of Biological & Biomedical Sciences, Aga Khan University, Karachi, Pakistan

**Keywords:** COVID-19, Outbreak of Corona Virus, Psychological health, Coping Strategies, Health care Professionals, Front liners

## Abstract

**Objective:**

This study was conducted to explore factors that can impact psychological health and coping strategies to help health care professionals (HCPs) to perform their duties.

**Methods:**

A cross sectional survey was conducted using structured questionnaire electronically shared with the participants after ethical approval. Descriptive statistics were calculated for socio demographic variables. Chi squared χ^2^ test was used to compare the responses between different groups of HCPs.

**Results:**

Survey was completed by 250 participants. They performed their duties diligently during outbreak but were concerned about their safety, had fear of infecting themselves and their family members. Lack of evidence-based guidelines for patient management, news about pandemic situation through media and to deal with uncooperative patients not willing for quarantine added to their stress. receiving honour and respect from general public in recognition of services, monetary benefit, adequate training to treat COVID-19, provision of personal protective equipment from government were reported as coping strategies for psychological impact.

**Conclusions:**

COVID-19 outbreak had psychological impact on HCPs, yet they continued to perform their duties carefully as moral obligation. Continued moral with financial support and acknowledgement of their services by government, organization and general public was reported to have psychological benefit.

## INTRODUCTION

Since the report of first cluster of COVID -19 (CORONA Virus) cases in December 2019, it has shown rapid spread over short span of time.^1^ WHO labelled virus as ‘severe acute respiratory tract coronavirus-2’ (SARS-CoV-2 / 2019-nCOV) and ‘COVID-19’ as global pandemic.^2^,^3^ Since then as per WHO report , more than 2.7 Million cases have been reported worldwide and 7,22,285 people have lost their lives.^4^ In Pakistan by August 10^th^ 2020 2,84,660 cases have been reported with around 6097 deaths.^5^

During these circumstances, healthcare professionals (HCPs) working as frontline soldiers are facing a multitude of challenges. Fear of getting infected themselves and their family members, increased workload, perplexing news about pandemic on social media, lack of evidence-based guidelines for patient management and inadequate provision of personal protective equipment (PPEs) are the factors that act as stressors.^6^-^9^ The physical and psychological stress continue to rise every passing day with increase in number of patients detected positive and contradicting reports.^10^,^11^

Various coping strategies proven successful from previous experience include provision of adequate PPEs, self-protection, infection prevention and control guidelines, institutional policies and SOPs based on evidence based practices.^12^,^13^ Acknowledgment and gratitude by government, health care authorities, hospital administration, community, society and general public is also documented as effective coping strategy in time of pandemic.^14^

This study marks the preliminary initiation of identifying underlying factors responsible for causation of stress and strategies deemed necessary to cope with them. This will provide baseline data for multi-dimensional mental health dynamics and psychological interventions deemed necessary for frontline workforce to win this battle.

## METHODS

This cross-sectional survey was carried out after obtaining ethical approval from Institutional Review board of Rawalpindi Medical University, (Ref.No. 57/IREF\RMU\2020, Dated: 01-05-2020). Purposive sampling technique was employed and estimated sample size was 250 using 95% confidence level and 5% absolute precision, effect size of 50%, and 5% added for non-response. The included HCPs comprised of consultants, medical officers, faculty members, residents and house officers serving all over Pakistan. The online survey (google) Form was electronically shared and data was collected after obtaining informed consent. Only participant selecting the option ‘*Yes’* were directed towards next page and were given option to quit at any time.

### Study Questionnaire

It comprised of three sections; socio demographic profile of study participants, factors causing stress and coping strategies to reduce stress during COVID-19 respectively. Responses obtained on 5-point Likert scale ranging from 0-5(Strongly disagree, disagree, neutral, agree and strongly agree).

### Statistical Analysis

Data was analysed using SPSS version 23. Results were compared in sub categories of consultants, medical officers, faculty, resident and house officers. Descriptive statistics was used to present data as frequency and percentages. Responses were compared by chi square test, p-value of < 0.05 considered significant.

## RESULTS

Survey was completed by 250 participants. Characteristics of the study participants are provided in Supplementary Table. Factors that caused stress are shown in Table-I. Lack of evidence-based guidelines for patient management (p<0.001) was reported highest by consultants whereas distress from news through media was reported higher by the residents (p=0.002). Medical officers were concerned with uncooperative patients, not willing for quarantine (p<0.001).

**Table-I:** Factors causing stress among different groups of health care professionals.

Question	Response	Consultant 40 (16)	Medical officers 70 (28)	Faculty 53(21)	Resident 47(19)	House Officer 40(16)	p-value
Do you feel fear of getting infected?	Strongly disagree	11(27.5)	3(4.2)	33(47.1)	3(6.3)	8(20)	<0.001
	Disagree	5(12.5)	2(2.8)	11(20.7)	11(23)	8(20)	
	Neutral	22(55)	8(11.4)	2(3.7)	2(4.2)	14(35)	
	Agree	1(2.5)	22(31.4)	4(5.6)	5(10.6)	5(12.5)	
	Strongly agree	1(2.5)	35(50)	3(5.6)	26(55.3)	5(12.5)	
Seeing your colleagues getting infected	Strongly disagree	4(10)	7(10)	5(9.4)	3(6.3)	5(12.5)	0.57
	disagree	4(10)	8(11.4)	10(5.3)	5(10.6)	5(12.5)	
	Neutral	2(5)	15(21.4)	24(45.2)	25(10.6)	4(10)	
	Agree	6(15)	20(28.5)	11(20.7)	8(17)	8(20)	
	Strongly agree	24(60)	25(35.7)	3(5.6)	6(12.7)	18(45)	
Fear of bringing infection home and infecting family members	Strongly disagree	5(12.5)	3(4.2)	3(5.6)	5(10.6)	6(15)	<0.001
	disagree	4(10)	2(2.8)	5(9.4)	5(10.6)	7(17.5)	
	Neutral	20(50)	35(50)	5(9.4)	3(6.3)	15(37.5)	
	Agree	6(15)	8(11.4)	10(5.3)	7(14.8)	7(17.5)	
	Strongly agree	5(12.5)	22(31.4)	30(56.6)	27((57.4)	5(12.5)	
Feeling helpless in managing patients appropriately	Strongly disagree	1(2.5)	10(7)	3(5.6)	7(14.8)	10(25)	0.41
	disagree	1(2.5)	19(27.1)	6(11.3)	10(21.2)	8(20)	
	Neutral	2(5)	31(44.2)	4(5.6)	10(2.2)	13(32.5)	
	Agree	10(4)	4(5.7)	11(20.7)	13(27.6)	7(17.5)	
	Strongly agree	25(6.25)	6(8.5)	29(54.7)	3(6.3)	2(5)	
Seeing patients dying in front of you	Strongly disagree	1(2.5)	3(4.2)	5(9.4)	3(6.3)	8(20)	0.009
	disagree	1(2.5)	2(2.8)	10(5.3)	8(17)	8(20)	
	Neutral	1(2.5)	8(11.4)	24(45.2)	22(46.8)	15(37.5)	
	Agree	10(4)	20(28.5)	11(20.7)	8(17)	7(17.5)	
	Strongly agree	27(67.5)	37(52.8)	3(5.60	6(12.7)	2(5)	
Lack of evidence based guidelines for patient management	Strongly disagree	1(2.5)	10(7)	3(5.6)	3(6.2)	6(15)	<0.001
	disagree	3(7.5)	19(27.1)	5(9.4)	6(12.7)	10(25)	
	Neutral	1(2.5)	31(44.2)	5(9.4)	24(51)	12(30)	
	Agree	9(22.5)	4(5.7)	12(22.6)	8(17)	8(20)	
	Strongly agree	25(62.5)	6(8.5)	28(52.8)	6(12.7)	4(10)	
Workload has been increased due to increasing number of COVID-19 cases	Strongly disagree	5(12.5)	3(4.2)	5(9.4)	3(6.3)	8(20)	0.91
	disagree	4(10)	2(2.8)	10(5.3)	11(23.4)	7(17.5)	
	Neutral	20(50)	8(11.4)	24(45.2)	2(4.2)	15(37.5)	
	Agree	6(15)	20(28.5)	11(20.7)	5(10.6)	6(15)	
	Strongly agree	5(12.5)	37(52.8)	3(5.6)	26(55.3)	4(10)	
Increasing number of infected patients everyday	Strongly disagree	5(12.5)	3(4.2)	5(9.4)	3(6.3)	8(20)	5.31
	disagree	4(10)	2(2.8)	12(22.6)	8(17)	8(20)	
	Neutral	25(62.5)	8(11.4)	22(41.5)	5(10.6)	14(350	
	Agree	2(5)	22(31.4)	11(20.7)	7(14.8)	5(12.5)	
	Strongly agree	4(10)	35(50)	3(5.6)	24(45.2)	5(12.50	
News about pandemic situation on electronic and social media	Strongly disagree	11(27.5)	10(14.2)	3(5.6)	3(6.3)	5(12.5)	0.002
	disagree	5(12.5)	19(27.1)	5(9.4)	6(12.7)	5(12.5)	
	Neutral	18(45)	31(44.2)	5(9.4)	24(45.2)	4(10)	
	Agree	3(7.5)	4(5.7)	10(5.3)	8(17)	8(20)	
	Strongly agree	3(7.50	6(8.5)	30((56.6)	6(12.7)	18(45)	
Dealing with uncooperative patients who are not willing for quarantine	Strongly disagree	5(12.5)	3(4.2)	5(9.4)	3(6.3)	8(20)	<0.001
	disagree	4(10)	2(2.8)	12(22.6)	10(21.2)	8(20)	
	Neutral	21(52.5)	8(11.4)	22(41.5)	20(42.5)	15(37.5)	
	Agree	7(17.5)	21(30)	11(20.7)	8(17)	4(10)	
	Strongly agree	5(12.5)	36(51.4)	3(5.6)	6(12.7)	5(12.5)	
Extended duty hours due to COVID-19 outbreak	Strongly disagree	5(12.5)	3(4.2)	5(9.4)	3(6.3)	7(17.5)	4.14
	disagree	4(10)	2(2.8)	12(22.6)	11(23.4)	9(22.5)	
	Neutral	5(12.5)	8(11.4)	18(25.7)	4(8.5)	5(12.5)	
	Agree	9(22.5)	23(31.9)	10(14.2)	5(10.6)	5(12.5)	
	Strongly agree	19(47.5)	33(47.1)	7(10)	28(59.5)	16(40)	
Discomfort due to extended wearing of PPEs	Strongly disagree	5(12.5)	3(4.2)	7(10)	3(6.3)	8(20)	0.56
	Disagree	4(10)	2(2.8)	12(22.6)	9(19.1)	8(20)	
	Neutral	9(22.5)	8(11.4)	5(9.4)	2(4.2)	5(12.5)	
	Agree	9(22.5)	20(28.5)	9(16.9)	11(23.4)	5(12.5)	
	Strongly agree	15(37.5)	37(52.8)	20(37.7)	22(46.8)	14(35)	
Blaming on doctors from media	Strongly disagree	5(12.5)	10(14.2)	4(7.5)	3(6.3)	5(12.5)	0.51
	disagree	6(1.5)	19(27.1)	4(7.5)	8(17)	5(12.5)	
	Neutral	7(17.5)	6(8.5)	5(9.4)	5(10.6)	4(10)	
	Agree	8(20)	4(5.7)	12(22.6)	8(17)	8(20)	
	Strongly agree	16(40)	31(44.2)	28(52.8)	23(48.9)	18(45)	
Lack of Support and encouragement at workplace	Strongly disagree	1(2.5)	10(14.2)	4(7.5)	3(6.3)	5(12.5)	7.67
	disagree	3(7.5)	19(27.1)	4(7.5)	8(17)	6(15)	
	Neutral	1(2.5)	6(8.5)	5(9.4)	5(10.6)	5(12.5)	
	Agree	10(25)	4(5.7)	10(5.3)	6(12.7)	8(20)	
	Strongly agree	26(65)	31(44.2)	30(56.6)	25(53.1)	20(50)	

Responses are compared on basis of chi square test, p-value <0.05 considered significant*

Table-II compared coping strategies suggested by HCPs to alleviate stress during pandemic. Receiving honour and respect from general public was reported highest by faculty (p=0.03) and receiving remuneration from government by medical officers (p<0.001). Consultants mentioned monetary benefits and compensation to be provided by organisation (p=0.02). Provision of PPEs and adequate training to treat COVID-19 patients by organization (p=0.01) was recommended by all HCPs.

**Table-II:** Factors reducing stress among different groups of health care professionals during COVID-19 outbreak.

Question	Response	Consultant 40 (16)	Medical officers 70(28)	Faculty 53(21)	Resident 47(19)	House Officer 40(16)	p-value
Pakistan not showing rapid upsurge in COVID-19 cases	Strongly disagree	9(22.5)	5(7.1)	35(66)	3(4.2)	5(12.5)	<.001
	disagree	7(17.5)	2(2.8)	13(24.5)	9(19.1)	8(20)	
	Neutral	20(50)	6(8.5)	2(3.7)	4(8.5)	15(37.5)	
	Agree	2(5)	24(3.4)	4(5.6)	7(14.8)	6(15)	
	Strongly agree	1(2.5)	37(52.8)	3(4.6)	28(59.5)	6(15)	
Patients recovering from Illness	Strongly disagree	4(10)	7(10)	7(13.20	3(6.3)	5(12.5)	0.04
	disagree	4(10)	8(11.4)	12(22.6)	5(10.6)	5(12.5)	
	Neutral	4(10)	15(21.4)	22(41.5)	24(42.5)	4(10)	
	Agree	7(17.5)	21(30)	10(18.8)	9(19.1)	7(17.5)	
	Strongly agree	21(5.25)	24(34)	4(5.6)	6(12.7)	19(47.5)	
Seeing HCPs working diligently to provide best services	Strongly disagree	6(15)	3(4.2)	3(4.6)	5(10.6)	5(12.5)	0.09
	disagree	4(10)	3(4.2)	4(5.6)	4(8.5)	8(20)	
	Neutral	22(55)	36(51.4)	6(11.3)	4(8.5)	16(40)	
	Agree	4(10)	7(10)	11(20.7)	6(12.7)	6(15)	
	Strongly agree	4(10)	21(30)	28(52.8)	28(59.5)	5(12.5)	
Receiving honour and respect from general public	Strongly disagree	1(2.5)	10(7)	3(4.6)	7(14.8)	10(250	0.03
	disagree	2(5)	19(27.1`)	6(11.3)	11(23)	8((20)	
	Neutral	3(7.5)	30(42.8)	4(5.6)	9(19.1)	15(37.5)	
	Agree	9(22.5)	5(7.1)	10(18.8)	14(29.7)	5(12.5)	
	Strongly agree	24(6)	6(8.5)	30(56.6)	2(4.2)	2(5)	
Receiving regard and remuneration from government in recognition of services	Strongly disagree	1(2.5)	3(4.2)	5(9.4)	4(8.5)	8(20)	<0.001
	disagree	1(2.5)	2(2.8)	9(16.8)	8(17)	8(20)	
	Neutral	3(7.5)	10(7)	25(47.10	20(42.5)	16(40)	
	Agree	10(4)	20(28.5)	10(18.8)	8(17)	6(15)	
	Strongly agree	25(6.25)	35(50)	4(5.6)	7(14.8)	2(5)	
Monetary benefit, compensation provided by organisation	Strongly disagree	1(2.5)	10(7)	3(4.6)	4(8.5)	4(10)	0.02
	disagree	3(7.5)	20(28.5)	5(9.4)	6(12.7)	11(27.5)	
	Neutral	2(5)	30(42.8)	5(9.4)	23(48.90	13(32.5)	
	Agree	9(22.5)	4(5.7)	13(24.5)	9(19.1)	8(20)	
	Strongly agree	24(60)	6(8.5)	27(50.9)	5(10.6)	4(10)	
Provision of PPEs & adequate training to treat COVID-19 patients	Strongly disagree	5(12.5)	3(4.2)	5(9.4)	3(6.3)	8(20)	0.01
	disagree	4(10)	2(2.8)	10(18.8)	10(21.2)	8(20)	
	Neutral	19(47.5)	7(10)	23(43.3)	3(6.3)	13(32.50	
	Agree	7(17.5)	22(31.4)	12(22.6)	5(10.6)	5(12.5)	
	Strongly agree	5(12.5)	36(51.4)	3(4.6)	26(55.3)	5 (12.5)	

Responses are compared on basis of chi square test, p-value <0.05 considered significant *

## DISCUSSION

Evidence from previous epidemics namely severe acute respiratory syndrome (SARS) and Middle East respiratory syndrome (MERS) has suggested that these out breaks had significant impact on psycho social well-being of frontline workers.^15^ Results of our study suggest that HCPs suffered a considerable psychological distress during the pandemic and main stressors reported were fear of getting infected and infecting family members similar to findings of recent study conducted in China.^16^ Comparable findings are reported from multicentre, multinational studies conducted during COVID -19.^17^

Lack of evidence-based guidelines for patient management was reported by consultants and faculty since there is no definite treatment and vaccine available.^18^,^19^ News about pandemic situation on electronic and social media was also reported as a stressor. This phenomenon is labelled as “Infodemic” meaning that excessive amount of misinformation that is real but inaccurate, unauthentic and not supported by scientific evidence.^20^ Another stressor reported by medical officers was uncooperative attitude of patients during quarantine which is also supported by literature.^21^

HCPs were satisfied with their duty hours, extended wearing of PPEs, level of support and encouragement provided by government consistent with results of study conducted in Hunan,China.^16^ Appreciation of HCPs as front liners in recent pandemic has been mentioned as a source of strong moral booster for frontliners.^22^ Furthermore, receiving regard and remuneration from government in recognition of services, monetary benefit, compensation provided by organisation were reported as stress relieving factors in our study. Recognition of services of HCPs can serve as a catalyst to motivate them to work and to fulfil their moral and social responsibilities and professional obligations diligently.

Our study though provided baseline data of possible causes of stress and effective coping strategies yet inclusion of nursing staff and allied health care should have been considered. In depth longitudinal studies by involving multiple health care centers are further suggested to enhance generalizability of results.

### Recommendations

Healthcare organizations and universities should organize virtual training courses for awareness about COVID 19 infection and prevention. Guidelines for control and prevention should be updated with sessions on hand washing techniques, donning & doffing and use of PPEs. Furthermore, telemedicine and telehealth consultation training courses should be encouraged.

## CONCLUSION

The study findings showed that significant factors associated with stress in HCPs were fear of getting infected and infecting family members, lack of evidence-based guidelines for patient’s management, exaggerated news about pandemic situation on electronic and social media and dealing with uncooperative patients who are not willing for quarantine. The strategies serving as motivational factors to reassure HCPs to continue to work during pandemic were; Pakistan not showing rapid upsurge in COVID-19 cases as compared to rest of the world, recovery of patients from Illness, receiving respect from general public, remuneration from government, compensation provided by organisation with adequate provision of PPEs and arranging training sessions for COVID-19 patient management.

### Author’s Contribution:

**Dr. Khola and Dr. Ashad Sabir** designed the study and performed statistical analysis.

**Dr. Rehana and Dr. Umar** took part in collection of data.

All the authors made substantial contribution in literature search, writing of the manuscript and revising it for critical evaluation.
